# Fractional CO2 Laser Versus Micro Needling Radiofrequency for Post Acne Scarring: A Meta‐Analysis of RCTs

**DOI:** 10.1111/jocd.70765

**Published:** 2026-03-12

**Authors:** Yahya Argobi, Faisal Tobeigei, Faris I. Alasiri

**Affiliations:** ^1^ Department of Dermatology, College of Medicine King Khalid University Abha Saudi Arabia; ^2^ Security Forces Hospitals Program General Directorate of Medical Services, Ministry of Interior Riyadh Saudi Arabia

## Abstract

**Background:**

Post‐acne scarring is a prevalent dermatological concern with significant impact on quality of life. Fractional CO2 laser (FCL) and microneedling radiofrequency (MNRF) are widely used treatments, but their comparative efficacy and safety require further synthesis.

**Aims and Objectives:**

This meta‐analysis was conducted to compare the efficacy and safety of FCL versus MNRF in the management of post‐acne scarring.

**Methods:**

The analysis included eight randomized controlled trials involving a total of 249 patients. The primary outcomes evaluated encompassed improvement in acne scars, patient satisfaction scores, pain levels, the risk of post‐inflammatory hyperpigmentation, and the duration of erythema.

**Results:**

FCL demonstrated superior improvement in acne scars (MD: 0.31, 95% CI, 0.13–0.48, *p* = 0.0005) and higher patient satisfaction (MD: 0.32, 95% CI, 0.10–0.44, *p* = 0.005) compared to MNRF. However, FCL was associated with significantly higher pain scores (MD: 2.14, 95% CI, 1.90–2.37, *p* < 0.00001), an increased risk of PIH (RR: 4.44, 95% CI, 2.39–8.26, *p* < 0.00001), and a longer duration of erythema (MD: 1.72 days, 95% CI, 1.43–2.02, *p* < 0.00001). MNRF exhibited a more favorable safety profile.

**Conclusion:**

While FCL offers superior efficacy for post‐acne scarring, MNRF provides better tolerability. The choice of treatment should be individualized, considering patient‐specific factors such as skin type, pain tolerance, and recovery expectations. Further research with longer follow‐up and standardized outcomes is needed.

## 1 Introduction

Acne vulgaris is recognized as a chronic inflammatory dermatological condition that affects approximately 85% of the adult population worldwide [1]. The onset of acne vulgaris predominantly occurs during puberty, affecting 93% of late teenagers and 27% of adolescents [2]. Furthermore, the incidence of acne varies across different geographical regions, influenced by environmental, lifestyle, and genetic factors [3]. Acne impacts individuals of all genders, and its prevalence and severity can differ across various age groups, potentially resulting in post‐acne scars. Post‐acne scarring is recognized as a common dermatological issue that influences not only psychosocial well‐being but also diminishes quality of life and may induce anxiety or depression among those affected [4]. Due to the differing morphology, depth, and severity of these scars, effective management has remained a clinical challenge for dermatologists [5].

Contemporary therapeutic approaches, including Microneedling Radiofrequency (MNRF) and Fractional CO2 Laser (FCL) [6, 7]. They have gained popularity in recent years owing to their effective outcomes in the management of atrophic acne scars [8, 9]. Fractional CO^2^ laser induces collagen remodeling and skin resurfacing via controlled thermal injury to the epidermis [10]. Despite its effectiveness, fractional CO^2^; laser (FCL) is frequently associated with notable adverse effects, including post‐inflammatory hyperpigmentation, pruritus, and procedural discomfort [11]. Conversely, microneedling radiofrequency (MNRF) administers radiofrequency energy into the dermis using insulated microneedles, avoiding damage to the dermis [12]. Consequently, MNRF offers a superior safety profile while effectively stimulating collagen, thereby reducing recovery time.

Furthermore, the selection of a treatment among these two therapeutic strategies may hinge upon numerous factors, including skin type, scar topography, tolerance for downtime, and access to treatment or availability of equipment in nearby clinical facilities [13, 14]. Importantly, populations possessing Fitzpatrick skin types or darker skin tones may be at a heightened risk of adverse effects following CO2 laser treatment, indicating that Microwave Non‐Ablative Radio Frequency (MNRF) may serve as a viable alternative in specific cases [15]. Additionally, MNRF devices have advanced to incorporate variable depth needles and both insulated and non‐insulated tips, thereby enhancing their clinical utility.

Conversely, numerous randomized controlled trials (RCTs) have assessed the relative efficacy of these two interventions in the management of post‐acne scars [16, 17, 18]. Nonetheless, ongoing clinical uncertainty persists regarding which modality yields superior outcomes in terms of efficacy, safety, and patient satisfaction. Furthermore, no comprehensive study has been conducted to evaluate the comparative effectiveness of resolving inconsistencies within the literature and to determine whether FCL or MNRF provides better overall results for post‐acne scarring. Although individual trials may indicate minimal differences in scar improvement or patient preference, these findings are frequently constrained by small sample sizes, varying outcome metrics, heterogeneity in skin types, and disparities in treatment sessions or laser/MNRF parameters [13]. Therefore, a systematic aggregation of evidence employing meta‐analytical techniques is required to deliver a more robust and generalizable conclusion. Consequently, this study aims to comparatively assess the outcomes of Fractional CO2 Laser versus Microneedling Radiofrequency for Post‐Acne Scarring through a meta‐analysis of randomized controlled trials. The findings also delineate existing knowledge gaps, promote personalized treatment choices based on scar type and skin attributes, and guide future clinical research trajectories in aesthetic dermatology.

## 2 Methods

### 2.1 Search Design

This meta‐analysis was conducted in accordance with the “Reporting Items for Systematic Review and Meta‐Analysis (PRISMA)” guidelines [19]. There are no additional ethical review requirements, as the study involves previously published randomized controlled trials. The study was registered on PROSPERO database 2025, CRD420251112929.

### 2.2 PICO Framework

This study used the Population Intervention Control Outcome (PICO) framework to guide the search [20].

**Population:** Patients of any age or gender diagnosed with post‐acne atrophic scarring (e.g., icepick, rolling, or boxcar scars), regardless of skin type.

**Intervention:** Treatment involving the use of Fractional CO^2^ Laser (FCL) as a standalone therapy for post‐acne scarring.

**Comparator:** Treatment with Microneedling Radiofrequency (MNRF) as a standalone therapy.

**Primary Outcomes:** Improvement in acne scar severity (assessed using validated scales like the Goodman and Baron or ECCA), patient satisfaction scores, and the incidence of adverse effects (post‐inflammatory hyperpigmentation, erythema, pain, and downtime).

### 2.3 Search Strategy

Nine electronic databases, including PubMed/MEDLINE, EMBASE (Excerpta Medica Database), the Cochrane Central Register of Controlled Trials (CENTRAL), Web of Science, Scopus, ClinicalTrials.gov, Google Scholar, ScienceDirect, and CINAHL (Cumulative Index to Nursing and Allied Health Literature), were systematically searched for relevant articles from their inception to April 2025. The MeSH keywords employed for the retrieval of research articles from PubMed were (“Laser Therapy”[MeSH] OR “Carbon Dioxide”[MeSH] OR “Fractional CO2 Laser”) AND (“Microneedling”[MeSH] OR “Radiofrequency Therapy”[MeSH] OR “Microneedling Radiofrequency”) AND (“Acne Vulgaris”[MeSH] OR “Cicatrix”[MeSH] OR “Acne Scars”) AND (“Randomized Controlled Trials as Topic”[MeSH] OR “Randomized Controlled Trial”[Publication Type]) AND (“Meta‐Analysis as Topic”[MeSH] OR “Meta‐Analysis”[Publication Type]). A similar search methodology was applied across the other databases. The search was limited to articles published in the English language.

### 2.4 Eligibly Criteria

#### 2.4.1 Inclusion Criteria

1. RCTs including patients with post‐acne atrophic scarring.
2. Direct comparison of FCL monotherapy versus MNRF monotherapy.
3. Reporting of at least one primary outcome (scar improvement, patient satisfaction, or adverse effects).
4. Publication in English.

#### 2.4.2 Exclusion Criteria

Studies were excluded if they: (1) combined FCL or MNRF with other interventions (e.g., chemical peels, PRP); (2) involved non‐acne scars or other dermatological conditions; (3) were non‐randomized trials, reviews, case reports, or animal studies; or (4) did not report quantitative outcomes for meta‐analysis.

### 2.5 Data Extraction From Published Articles

The studies acquired through the database search were incorporated into the EndNote library. Subsequently, duplicate entries were eliminated. Reviewers independently applied the eligibility criteria to all individual studies in a blinded fashion. Data on demographic information—such as authors, publication year, country of origin, study population, study design, duration of follow‐up, and primary outcomes, including the improvement of acne scar severity, occurrence of adverse effects, and patient satisfaction scores—were systematically extracted. Any discrepancies were resolved through consultation with a third reviewer.

### 2.6 Risk Bias Assessment

The risk bias of the included randomized controlled trials (RCTs) was evaluated utilizing the Cochrane risk of bias methodology. Six domains—namely allocation concealment, participant blinding, selection bias, outcome assessment blinding, selective reporting, and additional bias—were employed to assess the potential risk of bias within the included studies. The scoring or categorization of each study was classified into three distinct levels: low risk, unclear, and high risk [21].

### 2.7 Statistical Analysis

Statistical analyses were conducted utilizing Review Manager Software (Cochrane Collaboration, version 5.4.0) [22]. For studies exhibiting potential heterogeneity, pooled data analysis was performed employing random‐effects models. A *p*‐value of less than 0.05 was considered statistically significant, serving as the threshold for significance. For continuous outcomes, including scar severity scores and patient satisfaction scores, mean differences (MDs) or standardized mean differences (SMDs) with 95% confidence intervals (CIs) were calculated. For dichotomous outcomes, such as the occurrence of adverse effects, risk ratios (RRs) or odds ratios (ORs) with 95% CIs were utilized. The I2 statistic was employed to evaluate heterogeneity, with I2 values exceeding 50% indicating substantial heterogeneity.

## 3 Results

### 3.1 Study Selection

**FIGURE 1:**
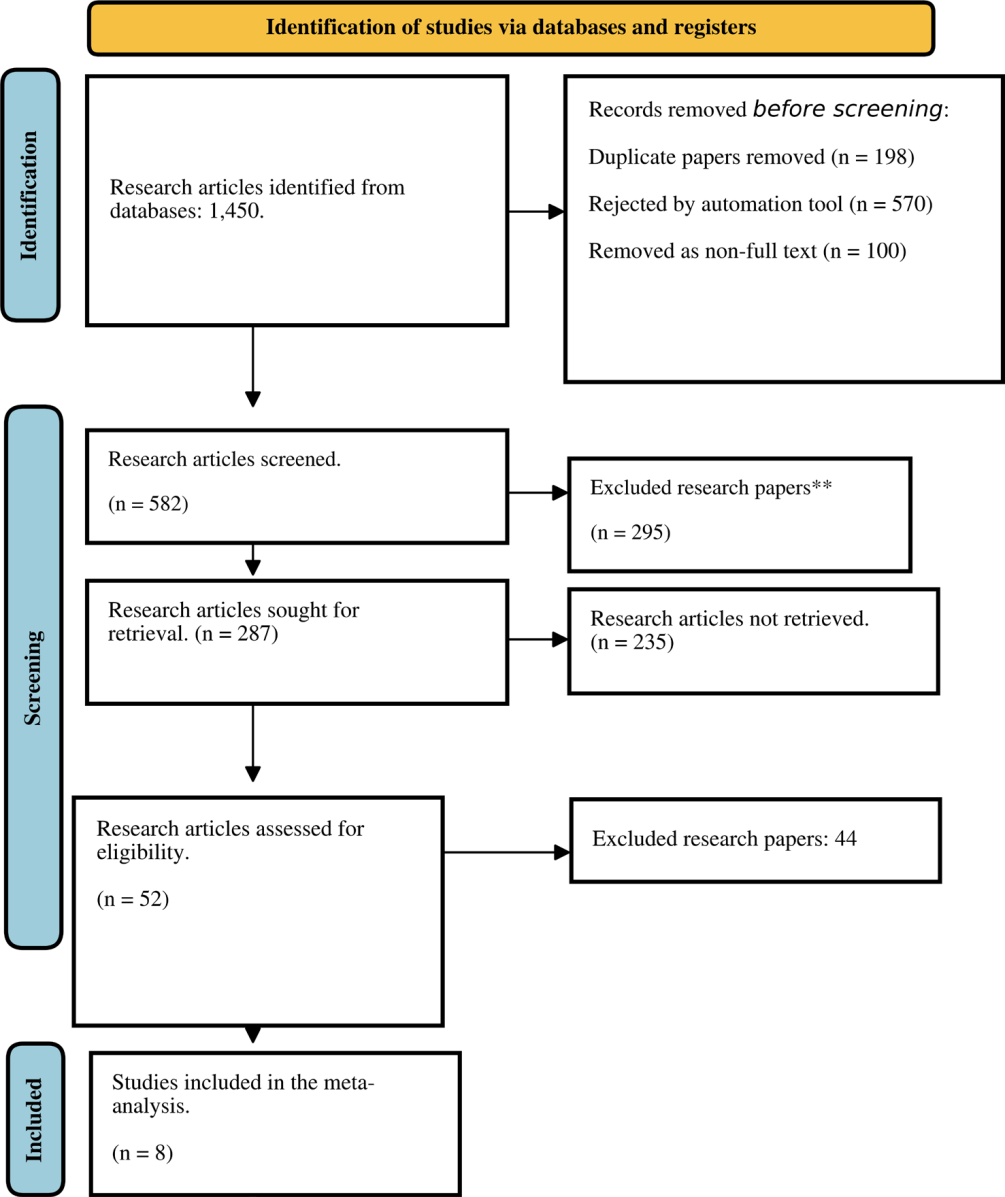
PRISMA Flow chart for screening and selection of included studies.

The selection and screening of research articles related to the study's aim were conducted in accordance with the PRISMA guidelines for this meta‐analysis. After database searches, 1450 records were generated, and 582 remained after removing duplicates and non‐full‐text records. These 582 articles were initially screened, resulting in 27 papers selected for retrieval. Only 52 papers were assessed for eligibility criteria, and the final inclusion consisted of eight research articles. Overall, this meta‐analysis is based on eight RCTs, as shown in Figure 1.

### 3.2 Characteristics of Included Studies

This study examined eight randomized and comparative clinical investigations assessing the efficacy and safety of Fractional CO^2^ Laser (FCL) in comparison to Micro‐needling Radiofrequency (MNRF) for the treatment of moderate to severe post‐acne scarring (Table 1). These investigations were conducted across various geographical regions, including India [16, 25, 27], China [23, 24], Pakistan [26], Iraq [22], and Denmark [17], with sample sizes ranging from 12 to 50 participants. The majority of trials employed split‐face or parallel group designs and conducted follow‐ups over approximately 2–3 months. Both interventions were evaluated for scar improvement utilizing the Physician's Global Assessment (PGA) or the Global Aesthetic Improvement Scale (GAIS). Patient satisfaction scores, measured on a Likert scale, were generally higher for FCL; however, pain scores, assessed with the Visual Analog Scale (VAS), were consistently lower for MNRF. Post‐inflammatory hyperpigmentation was more prevalent in the FCL cohort, while erythema persisted longer following FCL treatment in all studies reporting this outcome. Overall, MNRF exhibited better tolerability, whereas FCL demonstrated marginally higher efficacy in scar reduction in certain trials.

**TABLE 1:** Characteristics of included studies.

Author, year	Study population	Study groups	Country	Follow up	Study design	Mean reduction in acne scars by PGA or GAIS	Patient satisfaction Score (Likert scale)	Pain score (VAS, 0–10)	Post‐inflammatory hyper‐pigmentation	Erythema duration (days)
Hendel et al. 2023 [17]	15 patients with moderate to severe acne scars	F Co2 laser: 15 facial halves MNRF:15 facial halves	Denmark	3 months		PGAT1: 2.65 ± 0.67 T2: 2.20 ± 0.83	T1: 3.30 ± 0.73 T2: 3.0 ± 0.85	T1: 6.80 ± 0.70 T2: 4.25 ± 0.72	T1: 8 T2: 1	
Rajput et al., 2021 [16]	50 patients with moderate to severe acne scars (mean age: 2.6 years)	F Co2 laser:25 patients MNRF:25 patients	India	2 months	Prospective, randomized, open‐label	GAIST1: 1.82 ± 0.60 T2: 1.56 ± 0.65	T1: 4.8 ± 0.6 T2: 4.0 ± 0.7	T1: 5.8 ± 0.9 T2: 3.9 ± 1.1	T1: 7 T2: 1	T1: 3.2 ± 1.1 T2: 1.9 ± 0.7
Al Mallah et al., 2025 [22]	45 patients with moderate to severe acne scars (mean age: 28 years)	F Co2 laser: 15 patients MNRF:15 patients	Iraq	3 months	Open‐label, randomized controlled trial	GAIS T1: 1.4 ± 0.4 T2: 1.1 ± 0.3	T1: 3.8 ± 0.6 T2: 3.6 ± 0.5	T1: 5.9 ± 1.1 T2: 3.8 ± 0.8	T1: 5 T2: 1	
Le QU et al., 2025 [23]	12 patients with moderate to severe acne scars (mean age: 26.4 ± 4.1 years)	F Co2 laser: 12 facial halves MNRF:12 facial halves	China	3 months	Randomized, split‐face, pilot clinical study		T1: 3.0 ± 0.7 T2: 2.8 ± 0.6	T1: 6.1 ± 1.1 T2: 4.3 ± 0.9	T1: 4 T2: 1	T1: 4.2 ± 1.0 T2: 2.1 ± 0.7
Shin JU et al., 2012 [24]	25 patients with moderate to severe acne scars (Mean age: 24.8 ± 3.5 years)	F Co2 laser: 25 facial halves MNRF: 25 facial halves	China	3 months	Prospective, randomized, split‐face clinical trial		T1: 4.2 ± 0.6 T2: 4.0 ± 0.7	T1: 6.6 ± 0.8 T2: 4.4 ± 0.9	T1: 6 T2: 2	T1: 4.0 ± 0.9 T2: 2.2 ± 0.6
Sriram et al. 2024 [25]	32 patients with moderate to severe acne (mean age: 25.6 ± 3.9 years)	F Co2 laser:16 patients MNRF:16 patients	India	3 months	Retrospective comparative study	T1: 1.8 ± 0.6 T2: 1.5 ± 0.5	T1: 4.3 ± 0.7 T2: 4.1 ± 0.6	T1: 6.2 ± 1.0 T2: 4.3 ± 0.9	T1: 7 T2: 2	T1: 3.9 ± 0.8 T2: 2.1 ± 0.6
Obaid et al., 2021 [26]	40 patients with moderate to severe acne	F Co2 laser: 23 patients MNRF:17patients	Pakistan	3 months	Quasi‐experimental, parallel group				T1: 10 T2: 2	
Reddy et al., 2021 [27]	30 patients with moderate to severe acne scars	F Co2 laser: 15 patients MNRF:15 patients	India	3 months	Prospective, randomized comparative clinical trial				T1: 2 T2: 0	

Abbreviations: FCO^2^ laser, fractional CO^2^ laser; GAIS, global acne scarring classification score; MNRF, microneedling radiofrequency; PGA, physician's global assessment.

### 3.3 Risk Bias Assessment

**FIGURE 2:**
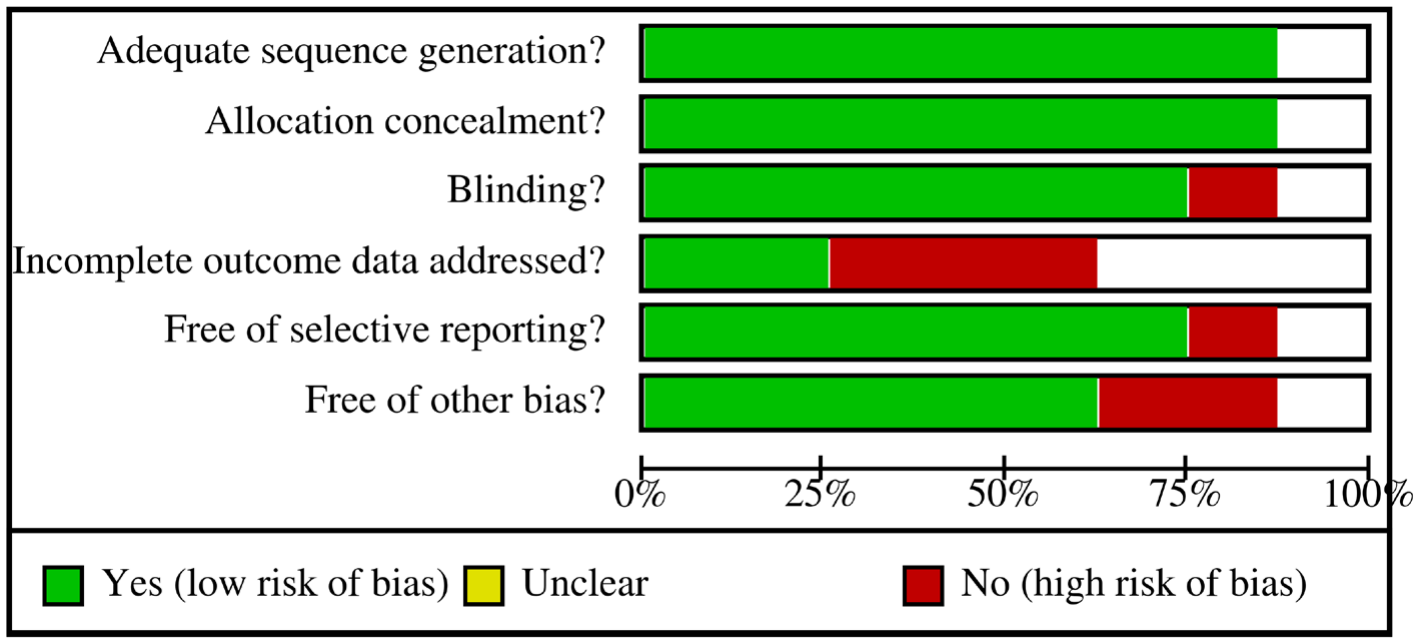
Summary of risk bias evaluations of included studies.

**FIGURE 3:**
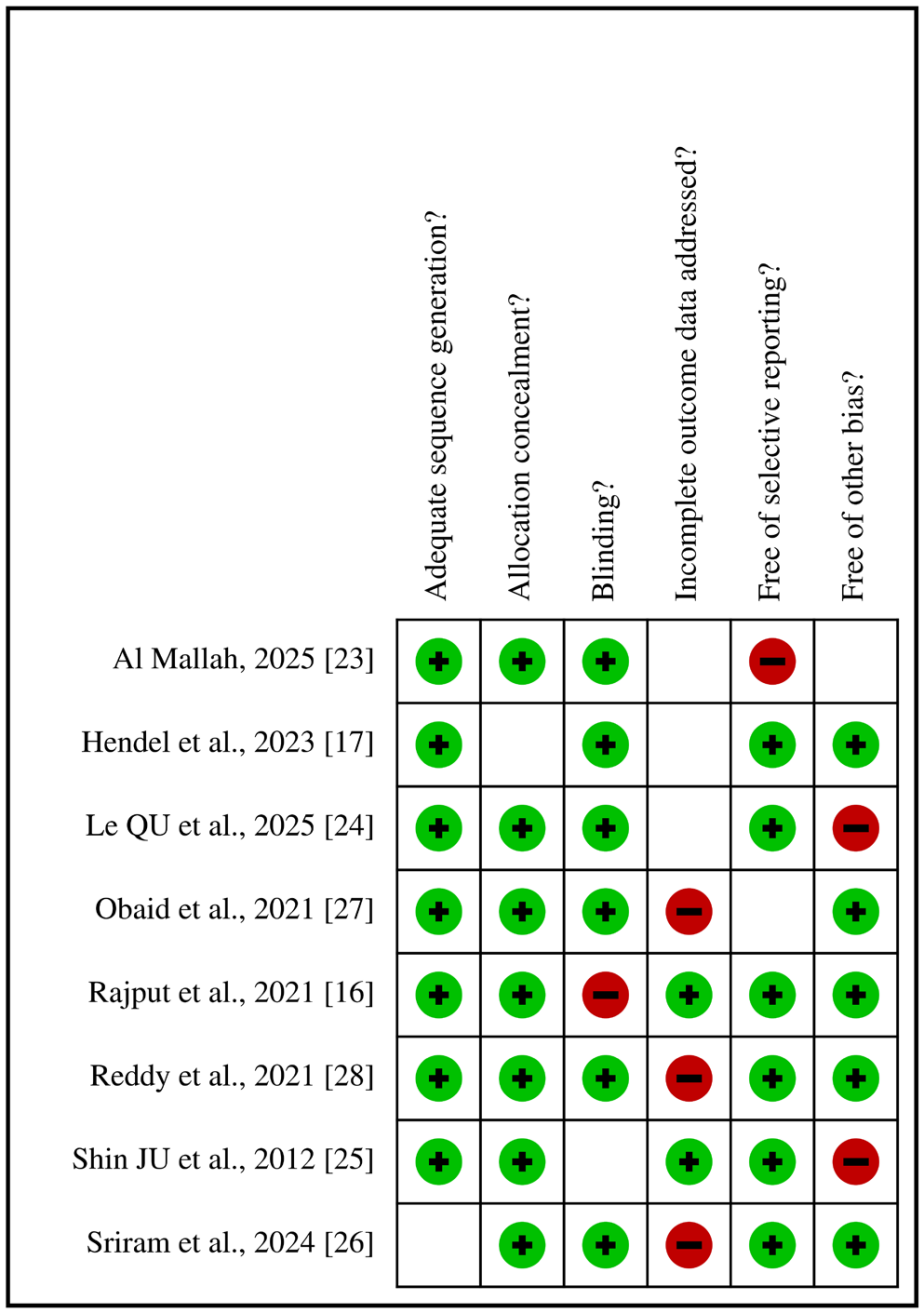
Results of risk bias evaluations of included studies.

The risk bias of the eight articles included was assessed in accordance with the systematic review guidelines outlined in the Cochrane Handbook (version 5.3). The findings were depicted through bias figures (Figures 2 and 3). The results indicated that all eight articles exhibited a low risk of bias, thereby satisfying the criteria for subsequent analysis.

### 3.4 Primary Outcomes

#### 3.4.1 Improvements in Acne Scars by Pga or Gais

**FIGURE 4:**
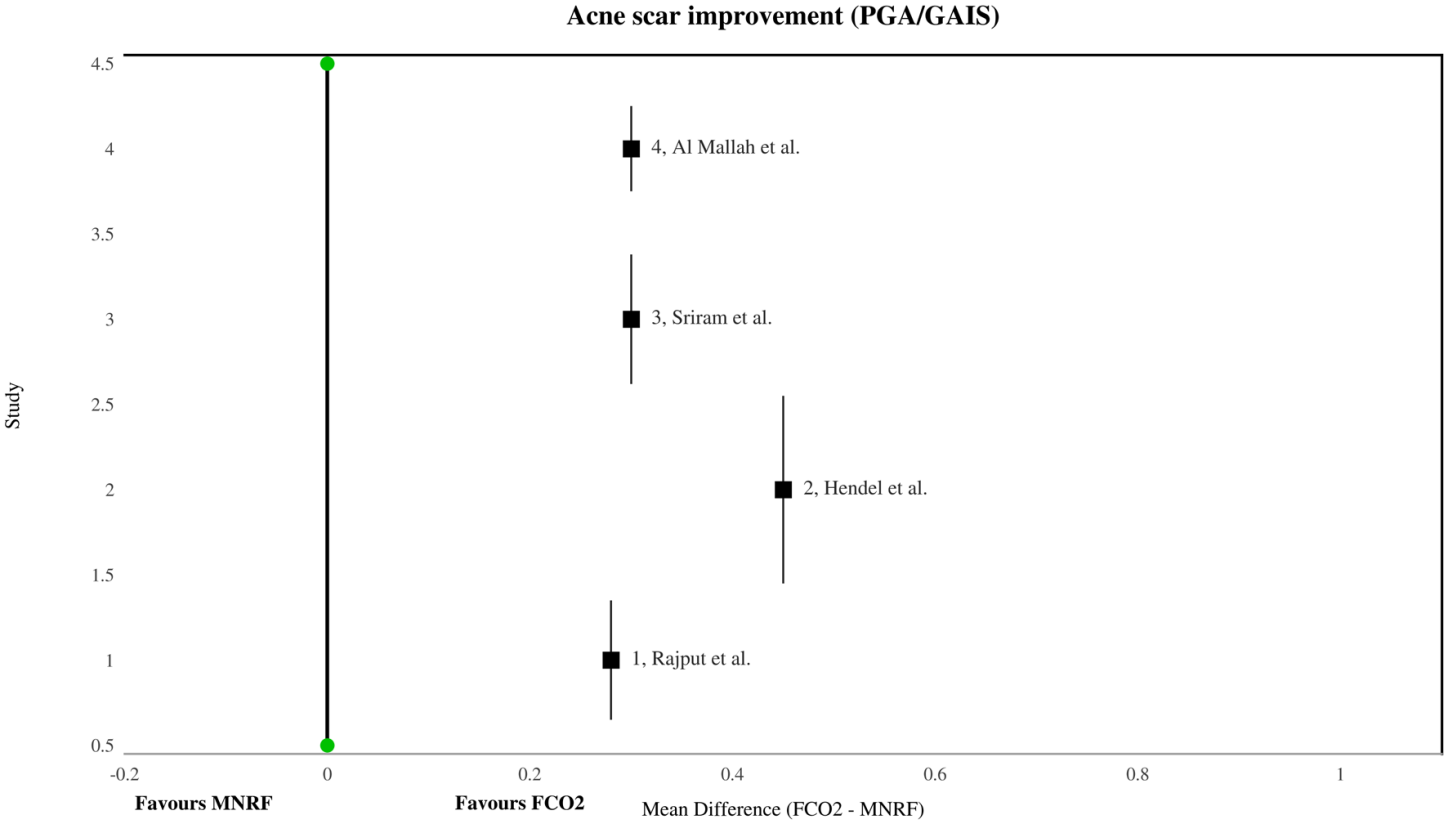
Forest plot showing the mean difference in acne scar improvement scores (PGA/GAIS) comparing Fractional CO^2^ Laser (FCO2) with Microneedling Radiofrequency (MNRF). Values to the right of the null line (0) favor FCO2, while values to the left favor MNRF.

**FIGURE 5:**
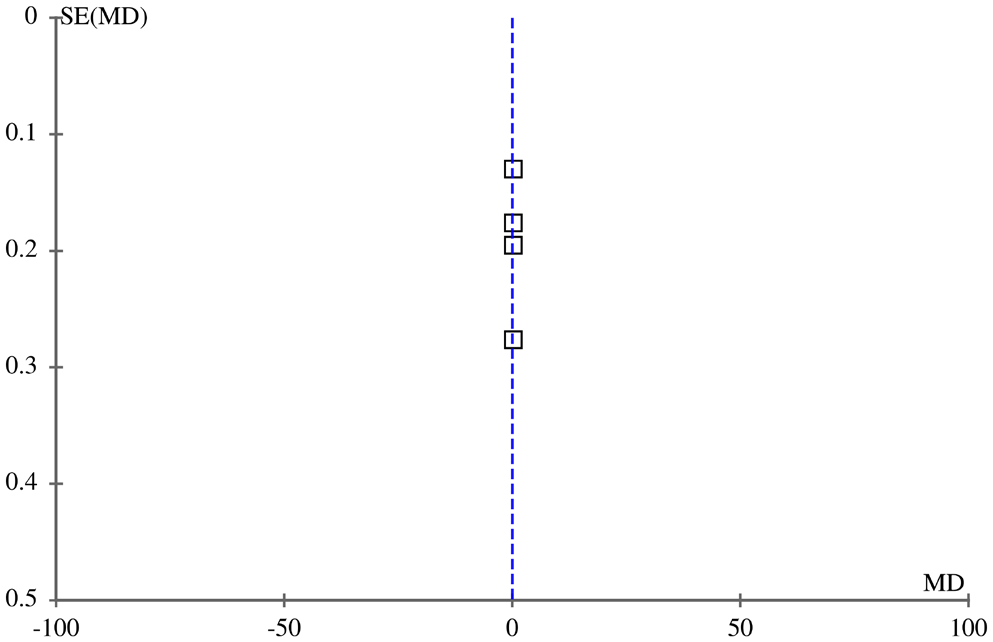
Funnel plot of mean difference of improvements in acne scar scores among groups receiving FCO2 and MNRF.

Four out of eight studies reported improvements or mean reductions in acne scars among patients with post‐acne scars following FCO2 laser and MNRF treatments. The pooled analysis demonstrated that post‐acne scars significantly improved after FCO2 compared to MNRF [MD: 0.31 (95% CI, 0.13–0.48), I2 = 0%, *p* = 0.0005], as illustrated in Figure 4. The symmetrical distribution of included studies on the funnel plot indicated low publication bias among the findings, as shown in Figure 5.

#### 3.4.2 Patient Satisfaction Score (Likert Scale)

**FIGURE 6:**
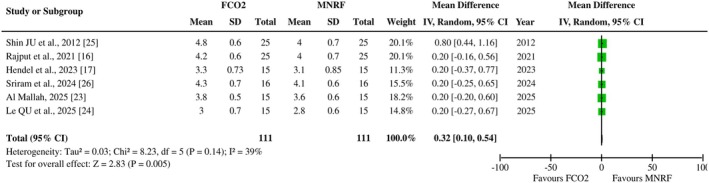
Forest plot of the mean difference of patients’ satisfaction scores among groups receiving FCO2 and MNRF.

**FIGURE 7:**
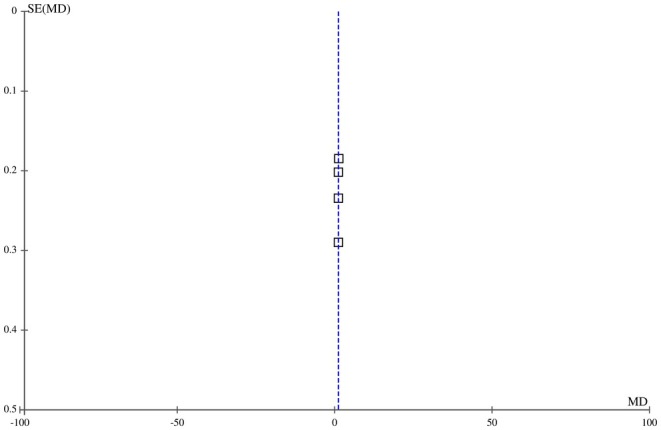
Funnel plot of the mean difference of patients’ satisfaction scores among groups receiving FCO2 and MNRF.

Six out of eight included articles reported patient satisfaction scores as an outcome among the cohort with post‐acne scars receiving FCO2 laser and MNRF as therapeutic interventions. The pooled analysis demonstrated a significant improvement in patient satisfaction scores (measured using a Likert scale) following FCO2 treatment in comparison to MNRF [MD: 0.32 (95% CI, 0.10–0.44), I2 = 39%, *p* = 0.005], as illustrated in Figure 6. The symmetrical distribution observed in the funnel plot of the included studies suggests minimal publication bias within the findings, as depicted in Figure 7.

#### 3.4.3 Pain Score (VAS, 0–10)

**FIGURE 8:**
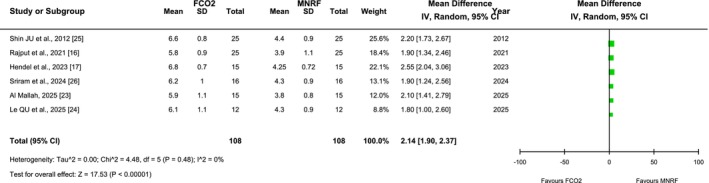
Forest plot of the mean difference of pain scores among groups receiving FCO2 and MNRF.

**FIGURE 9:**
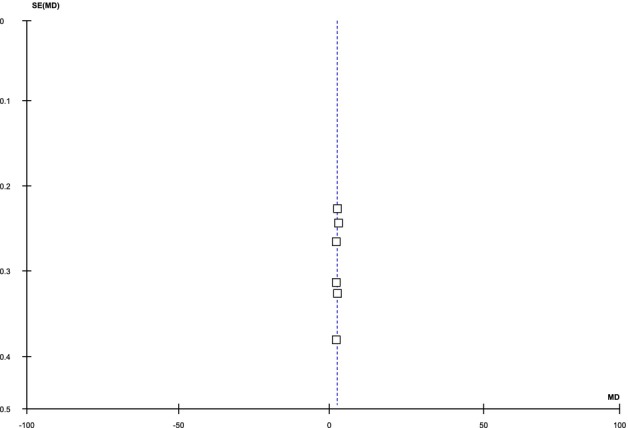
Funnel plot of the mean difference of pain scores among groups receiving FCO2 and MNRF.

Six out of eight included articles reported the pain scores as an outcome among the patient cohort with post‐acne scars receiving FCO2 laser and MNRF as therapeutic interventions. The pooled analysis indicated that pain scores, measured by the Visual Analog Scale (VAS), were significantly higher in the group receiving FCO2 compared to MNRF [MD: 2.14 (95% CI, 0.19–2.37), I2 = 0%, *p* < 0.00001], as illustrated in Figure 8. The symmetrical distribution of the included studies on the funnel plot suggested a low risk of publication bias among the studies, as depicted in Figure 9.

#### 3.4.4 Post‐Inflammatory Hyperpigmentation (PIH)

**FIGURE 10:**
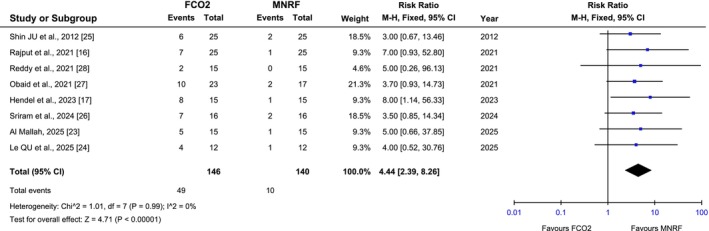
Forest plot of the Risk ratio of PIH among groups receiving FCO2 and MNRF.

**FIGURE 11:**
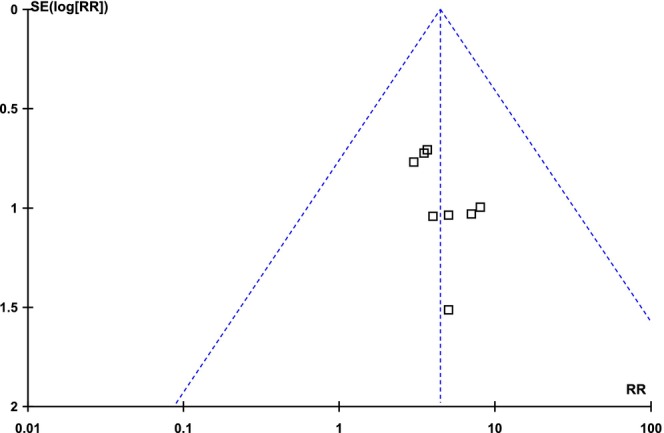
Funnel plot of the Risk ratio of PIH among groups receiving FCO2 and MNRF.

All included articles reported the incidence of PIH. The pooled analysis demonstrated that the incidence of PIH was significantly higher in the FCL group compared to MNRF [RR: 4.44 (95% CI, 2.39–8.26), I2 = 0%, *p* < 0.00001], as illustrated in Figure 10. The symmetrical distribution on the funnel plot indicated low publication bias, as shown in Figure 11.

#### 3.4.5 Erythema Duration (Days)

**FIGURE 12:**
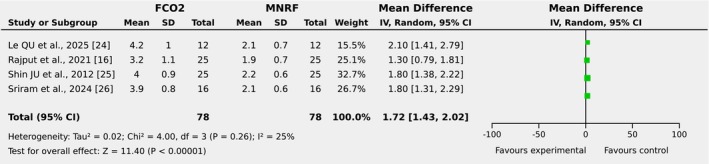
Forest plot of mean difference of erythema duration (days) among groups receiving FCO2 and MNRF.

**FIGURE 13:**
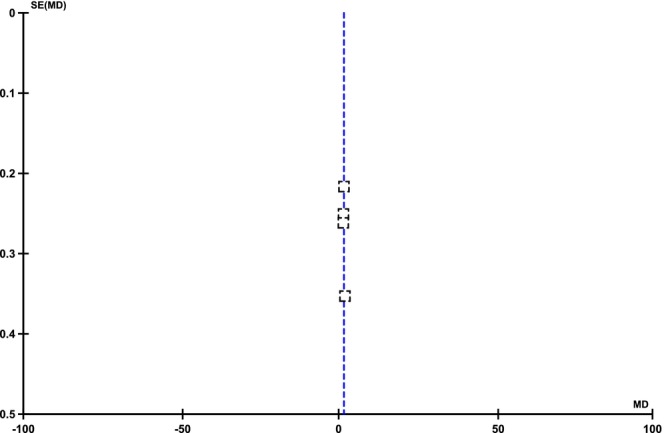
Funnel plot of mean difference of erythema duration (days) among groups receiving FCO2 and MNRF.

Four of the eight included articles reported the duration of erythema in days as an outcome among the patient group with post‐acne scars receiving FCO2 laser and MNRF as therapeutic strategies. The pooled analysis demonstrated that the duration of erythema (in days) was significantly greater in the group receiving FCO2 laser compared to MNRF [MD: 1.72 days (95% CI, 14–2), I2 = 25%, *p* < 0.00001], as illustrated in Figure 12. The symmetrical distribution observed in the funnel plot of the included studies indicated a low level of publication bias across the findings, as depicted in Figure 13.

## 4 Discussion

This meta‐analysis of 8 RCTs provides a comparative evaluation of FCL and MNRF for post‐acne scarring. The findings indicate that FCL offers superior efficacy in terms of scar improvement (MD: 0.31, *p* = 0.0005) and patient satisfaction (MD: 0.32, *p* = 0.005). This enhanced efficacy is likely attributable to the ablative nature of FCL, which promotes more aggressive collagen remodeling and skin resurfacing. However, this comes at the cost of a significantly less favorable safety profile, with higher pain scores (MD: 2.14, *p* < 0.00001), a substantially increased risk of PIH (RR: 4.44, *p* < 0.00001), and a longer duration of erythema (MD: 1.72 days, *p* < 0.00001). In contrast, MNRF demonstrated a more favorable safety and tolerability profile, as it delivers radiofrequency energy directly to the dermis while sparing the epidermis, thereby minimizing damage and associated adverse events.

The low to moderate heterogeneity (I^2^ 0%–39%) across outcomes strengthens the reliability of these pooled estimates. This consistency is likely due to the inclusion of only RCTs, which standardize comparisons. However, the absence of subgroup analyses means we cannot definitively account for the potential influence of variables such as Fitzpatrick skin type, specific scar morphology, or variations in device parameters (e.g., laser energy, microneedle depth). Future meta‐analyses with access to individual patient data would be valuable to explore these sources of clinical heterogeneity.

These findings were consistent with previous studies that reported the effective outcomes of ultra‐pulse fractional CO2 laser treatment (as monotherapy), including skin smoothness and the amelioration of acne scars, which are employed in the treatment of depressed and active acne scars [28, 29]. Furthermore, a separate study demonstrated the efficacy of microneedling radiofrequency in the treatment of acne scars through 12 studies [30]. However, no previous study has conducted a meta‐analysis comparing the outcomes of FCO2 laser and microneedling radiofrequency.

These findings underscore the importance of personalized treatment options from a clinical perspective. Healthcare professionals should exercise caution when suggesting the FCO^2^ laser to patients prone to hyperpigmentation or those with lower pain and downtime thresholds, despite its potential suitability for individuals seeking faster and more pronounced results [31]. Although the improvements may be minimally perceptible, MNRF presents a well‐tolerated alternative with fewer adverse effects, and many patients may consider it more advantageous owing to its overall balance between efficacy and tolerability [32]. To enhance patient satisfaction and facilitate collaborative decision‐making, dermatologists, cosmetic surgeons, and aesthetic practitioners may find these findings beneficial.

This meta‐analysis has several limitations. First, the reliance on subjective, clinician‐rated scales (PGA/GAIS) for the primary efficacy outcome introduces potential for observer bias. Future studies should incorporate more objective measurement tools, such as biometric devices or standardized photographic analysis. Second, the relatively short follow‐up periods (2–3 months) in the included trials preclude assessment of long‐term outcomes, including the stability of cosmetic improvement, the permanence of PIH, or scar recurrence. Longer‐term studies are essential. Third, the geographic concentration of included studies in Asia and the Middle East may limit the generalizability of the findings to other ethnic populations, particularly those of African or Caucasian descent with different skin characteristics and healing responses. Finally, the lack of blinding in some trials and inconsistent reporting of patient‐reported outcomes like quality of life and detailed downtime data further complicates a comprehensive comparison.

## 5 Conclusion

Overall, the findings of this study indicated that both FCL and MNRF are efficacious interventions for ameliorating post‐acne scarring, with FCL demonstrating marginally superior efficacy in scar improvement and patient satisfaction. Nevertheless, it is associated with increased discomfort, pigmentation alterations, and erythema. Clinicians should carefully weigh these benefits and risks during treatment planning, considering individual patient profiles, skin types, and preferences. Future research should prioritize larger, multicenter RCTs with prolonged follow‐up periods, standardized objective and patient‐reported outcome assessments, and predefined subgroup analyses based on skin type and scar morphology. Incorporating cost‐effectiveness and quality‐of‐life metrics will further refine personalized treatment recommendations.

## Funding

This work was supported by King Khalid University's Deanship of Scientific Research, GRP/122/44.

## Ethics Statement

This meta‐analysis synthesizes data from previously published studies and did not involve direct interaction with human participants or animals.

## Consent

Informed consent was not required because the analysis used only published, de‐identified aggregate data.

## Conflicts of Interest

The authors declare no conflicts of interest.

## Data Availability

The data that support the findings of this study are available from the corresponding author upon reasonable request.
